# Relationship between obesity and serum resistin, apelin, and sterol regulatory element binding protein-1c levels: the changes in the analyte levels durin g weight loss in obese patients

**DOI:** 10.1590/1806-9282.20250751

**Published:** 2025-12-15

**Authors:** Sena Erduhan, Ebubekir Bakan, Nurcan Kiliç Baygutalp, Hamit Hakan Alp

**Affiliations:** 1 Ataturk University, Faculty of Medicine, Department of Biochemistry – Erzurum, Türkiye.; 2 Ataturk University, Faculty of Pharmacy, Department of Biochemistry – Erzurum, Türkiye.; 3 Van Yuzuncu Yil University, Faculty of Medicine, Department of Biochemistry – Van, Türkiye.

**Keywords:** Apelin, BMI, Obesity, Resistin, Sterol Regulatory Element Binding Protein 1, Human

## Abstract

**OBJECTIVE:**

In obesity, the fat storage in the body increases excessively and affects the whole metabolism.

**METHODS:**

Sixty-two obese patients were included in the study. The effect of weight loss, which was achieved with a 3-month diet/exercise program, on the following analytes was investigated; serum total cholesterol, high-density lipoprotein-cholesterol, low-density lipoprotein-cholesterol, triacylglycerol, triglycerides (TAG), glucose, resistin, apelin, and a transcription factor sterol regulatory element binding protein-1c, and blood hemoglobin A1c. Basal (prediet/exercise) levels of analytes were taken as control values, and they were compared to the postdiet/exercise levels.

**RESULTS:**

After the 3-month diet/exercise program, apelin, hemoglobin A1c, and triacylglycerol, triglycerides levels decreased compared to basal levels, and those of sterol regulatory element binding protein-1c, glucose, and low-density lipoprotein-cholesterol increased.

**CONCLUSION:**

Considering the insulin-like effects of apelin, high levels of apelin in prediet/exercise phase can be interpreted as a kind of resistance to apelin, and the decreased postdiet/exercise apelin levels as a kind of normalization of it. The decrease in hemoglobin A1c and triacylglycerol, triglycerides levels following weight loss can be appreciated. High basal serum sterol regulatory element binding protein-1c levels suggest an increase in lipogenesis. The increases in glucose and low-density lipoprotein-cholesterol after diet/exercise program remain in a physiological range. However, we think that a longer diet/exercise follow-up in a larger population will further elaborate on the issue.

## INTRODUCTION

Adipocytes are involved in the synthesis and release of numerous enzymes, hormones, growth factors, and cytokines that are effective in energy homeostasis. These factors are called adipokines^1^.

Resistin is a cysteine-rich proinflammatory molecule whose main source is adipocytes in rodents and peripheral blood mononuclear cells (PBMCs) in humans^2^. Insulin resistance develops by deteriorating glucose homeostasis in mice treated with resistin, and administration of resistin antibodies in mice with dietary obesity reduces blood glucose levels and increases insulin sensitivity^3^. Considering the effects of resistin on inflammatory response and insulin resistance, it becomes important to investigate its role in obesity^4^. However, the relationship between weight loss and serum resistin level in obese patients is not clear because the major source of resistin in human blood circulation is not adipose tissue^5^.

In humans, apelin is secreted from endothelial cells, cardiac and skeletal muscle cells, and the brain, as well as adipose tissue^6^. This cytokine is also effective on body fluid homeostasis, blood pressure, angiogenesis, and energy metabolism^7^. Plasma insulin and apelin levels are high together in obese individuals. In addition, mRNA levels of apelin increase by ex vivo exposure of human subcutaneous adipose tissue to insulin^8^. Apelin synthesis is regulated by insulin, and apelin increases insulin sensitivity^8,9^. The increase in apelin production in obesity can be interpreted as one of the last protective mechanisms against obesity and the diseases it causes^10^.

Sterol regulatory element binding proteins (SREBPs) are transcription factors associated with lipogenesis, adipocyte development, and cholesterol homeostasis. SREBP-1c is the most produced isoform in human white adipose tissue cells^11^. SREBP-1c controls the expression of genes involved in fatty acid synthesis and plays a role in the differentiation of adipocytes and in the effects of insulin on carbohydrate and lipid metabolism. In obesity and type 2 diabetes mellitus (T2DM), insulin cannot suppress glucose production in the liver, and a condition called T2DM characterized by insulin resistance occurs. Similarly, while the effect of insulin on lipid synthesis is expected to decrease, paradoxically, the increase in lipogenesis is remarkable.

We aimed to analyze the levels of serum resistin, apelin, and SREBP-1c as well as serum total cholesterol, LDL-cholesterol, HDL-cholesterol, triacylglycerol, triglycerides (TAG), glucose, and blood glycated hemoglobin A1c (HbA1c) levels in varying degrees of obesity and to investigate the changes of these analytes by applying a 3-month diet/exercise program to obese individuals.

## METHODS

### Ethics approval

The study was approved by the Atatürk University Faculty of Medicine Clinical Research Ethics Committee, dated 15/04/2021 and numbered 2021.03.59. Male and female participants between the ages of 18–65 years were included in the study. The study did not include pediatric participants. The study was conducted in accordance with the Declaration of Helsinki and followed the ethical standards of the country. The study was conducted and reported in accordance with the STROBE (Strengthening the Reporting of Observational Studies in Epidemiology) guidelines^12^. All anonymized raw data, the data dictionary, and analysis scripts are available at Mendeley Data (doi: 10.17632/jrynm735yj.1). Access: open.

### Selection of obese individuals and diet/exercise program

Obese individuals were selected from the University's Obesity Center to apply a diet/exercise program on a voluntary basis. Patients were given a diet (1,000–1,500 kcal/day) and an exercise (at least 5,000 steps/day) program suitable for age, gender, and body mass index (BMI) parameters. After the 3-month diet/exercise program, the weights of the obese subjects were remeasured, and second samples (postdiet/exercise samples) were taken for the measurement of the same analytes. Those who lost less than 5% of their weight measured at the first examination were not included in the study. A total of 62 obese patients included in the study were divided into three subgroups based on their BMI values at their first admission. A BMI of more than 30 kg/m^2^ is considered obese^13^. The first-degree obese group (G1) included 19 individuals with a BMI of 30–34.9 kg/m^2^, the second-degree included (G2) 19 individuals with a BMI of 35–39.9 kg/m^2^, and the third-degree obese (morbid obese) (G3) included 24 subjects with a BMI ≥40 kg/m^2^.

A post-hoc power analysis using G*Power software (ver. 3.1.9.7) was performed to determine the achieved power of the study. Program inputs were test family: F tests, statistical test: Anova: Repeated measures, within factors, type of power analysis: Posthoc, effect size: 0.25 (medium)^14^, α: 0.05, total sample size: 62, number of groups: 3, number of measurements: 2, correlation among rep measures: 0.5, nonsphericity correction ε:1. Accordingly, the calculated achieved power was 97%.

### Analytical measurements

Serum total cholesterol, TAG, HDL-cholesterol, and LDL-cholesterol measurements were made by the photometric method on Beckman Coulter AU 5800 clinical chemistry autoanalyzer. Serum resistin, apelin, and SREBP-1c measurements were performed by the enzyme-linked immunosorbent assay (ELISA) method and according to the instructions included in commercial kits (Human Resistin ELISA Kit E0338Hu, Human Apelin ELISA Kit E2014Hu, Human Sterol regulatory element-binding protein 1C ELISA Kit E6666Hu, Bioassay Technology Laboratory, China). Performance characteristics of all ELISA kits are presented in Supplementary Table 1. HbA1c measurement in whole blood samples taken into hemogram tubes containing EDTA was performed by boronate affinity high-performance liquid chromatography (HPLC) method.

### Statistical analyses

Statistical analyses were performed using Analyze-it Software (Analyse-it Software, Ltd., Leeds, United Kingdom) and SPSS 23.0 package programs. The Kolmogorov-Smirnov test was used in the analysis of the normal distribution of continuous data. The Mann-Whitney U test was used in the comparative analysis of independent groups for data distributed non-normally. The Wilcoxon test was used for the comparison of dependent (repetitive) analyses. Since most of the data were not normally distributed in the normality test, the median and IQR (interquartile range) values were used for descriptive statistics.

## RESULTS

### Body weight loss (%)

Body weight loss (%) measurements of the obese group were repeated at the end of the 3-month diet/exercise program, and this group was divided into three subgroups based on their BMIs. Weight loss values of % according to their prediet/exercise body weights were calculated as an average of 8.6% for a total of 62 obese subjects. Among participants, 13 of the first-degree obese, 14 of the second-degree obese, and 17 of the third-degree obese lost weight between 5 and 9.9%. Those who lost more than 10% weight were six subjects in the first group, five in the second group, and seven in the third group.

### Biochemical results

Baseline characteristics are presented in Table 1. The cohort consisted predominantly of female participants, and the age distribution favored middle age. Gender and age distribution were similar among BMI subgroups, with no significant differences. However, consistent with the definition, BMI values showed a significantly increasing pattern across groups (Group I<Group II<Group III). All variables are reported as medians (IQR); different letters indicate statistically significant differences (p<0.05) between groups within the same row.

**Table 1:** The baseline characteristics of the participants.

	Obese cohort (n=62)		
Gender n (F/M)	58/4		
Age (year)	45.5 (38–55)		
BMI (kg/m^2^)	39.2 (34.2–43.3)		
	**Group I**	**Group II**	**Group II**
Gender n (F/M)	18/1^a^	23/1^a^	22/2^a^
Age (year)	44.3 (42.1–45.3)^a^	45 (43.2–46.4)^a^	45.4 (44.1–46.8)^a^
BMI (kg/m^2^)	32.1 (30.4–13.7)^a^	37 (36.1–39.2)^b^	42 (40.1–42.3)^c^

Different letters in the same row indicated a significant difference. BMI: body mass index.

Table 2 exhibits the statistical analysis of basal (first measurement) and postdiet/exercise (second measurement) levels of analytes in the obese group. As summarized in Table 2, significant changes were observed in some biochemical markers after the diet/exercise intervention. In the lipid profile, LDL-cholesterol levels increased significantly after the intervention, while TAG levels decreased significantly. No significant differences were observed between pre- and post-intervention in total cholesterol and HDL-cholesterol. Regarding glycemic indicators, fasting glucose slightly but statistically significantly increased after the intervention, while a small but significant decrease in HbA1c was observed. Adipose tissue apelin concentrations significantly decreased, while levels of the lipogenesis regulator SREBP-1c significantly increased. Resistin levels showed no significant changes. All variables are reported as medians (IQR) according to distributional characteristics, and comparisons are shown in Table 2.

**Table 2:** Basal (first measurement) and postdiet/exercise (second measurement) levels of analytes for all participants (n=62).

	First measurement median (IQR)	Second measurement median (IQR)	p
Total Chol (mg/dL)	197 (166–226)	201 (174–236)	0.28
HDL-Chol (mg/dL)	46.9 (41–53.4)	47 (41–56)	0.645
LDL-Chol (mg/dL)	117 (96–139)	128 (99–155)	↑**0.01** **
TAG (mg/dL)	138 (95–190)	126 (88–105)	↓**0.001** **
Glucose (mg/dL)	90 (84–103)	95 (86–105)	↑**0.016** *
HbA1c (%)	5.9 (5.5–6.1)	5.7 (5.5–6.02)	↓**0.045** *
Resistin (ng/L)	390 (298–573)	396 (302–651)	0.908
Apelin (ng/L)	225 (62–269)	98.2 (52.1–128.4)	↓**0.001** **
SREBP-1c (ng/mL)	4.18 (3.31–5.01)	4.5 (3.34–5.52)	↑**0.012** *

IQR: interquartile range; Total Chol: total cholesterol; HDL-Chol: high-density lipoprotein-cholesterol; LDL-Chol: low-density lipoprotein-cholesterol; TAG: triacylglycerol, triglycerides; HbA1c: hemoglobin A1c; SREBP-1c: sterol regulatory element binding protein-1c.

** p<0.01;

* p<0.05.;

Bold asterisk (*) means p<0.05, bold asterisk (**) means p<0.01.

Analyses performed according to obesity subgroups are presented in Table 3. In pre-post intervention comparisons, only apelin levels decreased significantly in the G1 (BMI 30-34.9) group. A significant decrease in HbA1c and apelin levels was observed in the G2 (BMI 35–39.9) group, while no significant changes were observed in other parameters. In the G3 (BMI ≥40) group, TAG and apelin levels decreased significantly. Although there was a trend toward a decrease in LDL-cholesterol and fasting glucose, the statistical threshold was not exceeded. HDL-cholesterol and SREBP-1c levels did not change significantly after the intervention in any group.

**Table 3:** Statistical analysis of obesity subgroups for basal (first measurement) and postdiet/exercise (second measurement) values.

		First measurement median (IQR)	Second measurement median (IQR)	P
GROUP-1 (BMI 30–34.9) (n=19)	Total Chol (mg/dL)	207.8 (180.4–235)	225.5 (188.8–255.8)^a^	0.303
	HDL-Chol (mg/dL)	49 (42.7–58.3)	47 (41.1–59.3)	0.345
	LDL-Chol (mg/dL)	125 (105–150)	142 (118.2–162)^b^	0.087
	TAG (mg/dL)	147 (89.3–178.3)	120 (83.3–176.1)	0.275
	Glucose (mg/dL)	88 (84.3–95.6)	95 (86.1–97)	0.181
	HbA1c (%)	5.7 (5.5–5.9)	5.7 (5.6–5.9)	0.782
	Resistin (ng/L)	350 (309–441)^e^	378 (283–433)^c^	0.524
	Apelin (ng/L)	103.3 (60.8–241.2)	92.4 (43.4–110.3)	↓**0.0092** **
	SREBP-1c (ng/mL)	3.98 (3.55–4.71)	4.12 (3.55–5.21)	0.246
GROUP-2 (BMI 35–39.9) (n=19)	Total Chol (mg/dL)	183.7 (164–229)	201 (157.2–263.3)	0.123
	HDL-Chol (mg/dL)	46 (36–50)	43 (38.5–54.2)	0.644
	LDL-Chol (mg/dL)	109 (92–138.7)	129 (92.5–171.7)	0.054
	TAG (mg/dL)	147 (95.5–190.8)	133 (101.3–175.5)	0.062
	Glucose (mg/dL)	92 (84.2–108.2)	99 (85.8–106.8)	0.418
	HbA1c (%)	5.9 (5.6–6.23)	5.7 (5.4–6.1)	↓**0.042** *
	Resistin (ng/L)	314 (283–369)	313 (269–361)^d^	0.252
	Apelin (ng/L)	155.6 (58.6–240.8)	72.3 (48.3–112.1)	↓**0.001** **
	SREBP-1c (ng/mL)	4.02 (2.35–4.73)	4.1 (3.17–5.07)	0.229
GROUP-3 (BMI ≥40) (n=24)	Total Chol (mg/dL)	195.2 (167.8–220)	189 (1,735–209.7)^a^	0.259
	HDL-Chol (mg/dL)	46.2 (42–53.2)	49 (42.4–56.2)	0.388
	LDL-Chol (mg/dL)	114 (91.9–129.7)	112.5 (99.4–134.5)^b^	0.056
	TAG (mg/dL)	131.5 (102–178.9)	121 (96.4–146.9)	↓**0.003** **
	Glucose (mg/dL)	90 (82.4–104.2)	92 (87.2–106.5)	0.063
	HbA1c (%)	5.9 (5.5–6.1)	5.8 (5.44–6.05)	0.329
	Resistin (ng/L)	541 (347–597)^e^	553 (369–704)^c^,^d^	0.076
	Apelin (ng/L)	265 (199–355)	107 (91–242)	↓**0.002** **
	SREBP-1c (ng/mL)	4.41 (3.95–4.94)	4.62 (3.09–4.99)	0.123

* p<0.05;

** p<0.01,

a In the second measurements, total cholesterol levels decreased significantly in the G3 group compared to the G1 group (p=0.03).

b In the second measurements, LDL levels decreased significantly in the G3 group compared to the G1 group (p=0.033).

c In the second measurements, resistin level was significantly higher in the G3 group than in the G1 group (p=0.022).

d In the second measurements, resistin level was significantly higher in the G3 group than in the G2 group (p=0.001).

e In the first measurements, resistin level was significantly higher in the G3 group than in the G2 group (p=0.003).

IQR: interquartile range; BMI: body mass index; Total Chol: total cholesterol; HDL-Chol: high-density lipoprotein-cholesterol; LDL-Chol: low-density lipoprotein-cholesterol; TAG: triacylglycerol, triglycerides; HbA1c: hemoglobin A1c; SREBP-1c: sterol regulatory element binding protein-1c. Bold asterisk (*) means p<0.05, bold asterisk (**) means p<0.01.

In between-group comparisons, at the second measurement time, total cholesterol and LDL-cholesterol levels were found to be lower in G3 than in G1. Regarding resistin, levels in G3 were higher than in G1 and G2 at the second measurement. Additionally, G3 had higher resistin levels than G2 at baseline.

In summary, the decrease in apelin levels after intervention was consistent across all BMI subgroups, while the improvement in TAG was evident only in the highest BMI group. Postintervention, atherogenic lipids (total cholesterol and LDL) were lower in G3 compared to G1, while resistin levels were higher in G3. All findings are reported as median (IQR), and statistical comparisons are presented in Table 3.

### Correlation analysis

**Figure 1:**
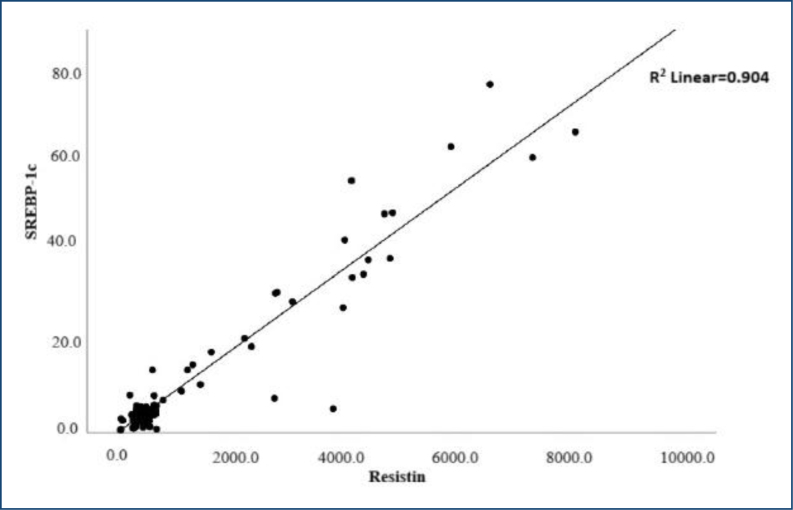
The correlation analysis between resistin and sterol regulatory element binding protein. Scatter dot representation.

Spearmen correlation analysis for some analytes, age, and BMI of obese subjects was performed to determine the relationships between parameters. For this analysis, postdiet/exercise levels of the obese group were recognized. The relations of resistin, apelin, and SREBP-1c to the other measurands were considered. There was a significant positive correlation between SREBP-1c and resistin (r=0.723, p<0.01) (Figure 1). In addition, we summarized all correlation analyses in Supplementary Table 2.

## DISCUSSION

This study aimed to determine the basal changes of some analytes and to compare them with the changes after diet/exercise program application for the obese. Globally, obesity places a heavy economic and health cost on people, healthcare systems, and societies^15^. Understanding the underlying mechanisms of obesity and its prevention is as important as its treatment. Our study contributes to the scientific literature, explaining how obesity, which is a predisposing to many diseases, responds metabolically through physical activity, diet, and resultantly the weight loss.

The statistically significant changes between the first and the second measurements in the obese include increased LDL-cholesterol, glucose, and SREBP-1c levels, and decreased HbA1c, apelin, and TAG levels in post-diet/exercise period. This situation is expected after diet/exercise. The increase in LDL-cholesterol and glucose in post-diet/exercise period remain in a physiological range. Two adipokines and one transcription factor measured in the study are discussed below. First of all, resistin and apelin may be considered as having antagonistic action to each other on metabolism via insulin with respect to lipid metabolism. Similarly, the contributory effect of insulin on the action mechanism of SREBP-1c is important in lipogenesis, as mentioned earlier. We selected two adipokines and one transcription factor in the study, which may be critical in the development of obesity.

Resistin is an adipokine known to promote insulin resistance. In our study, serum resistin levels did not significantly change after 3 months of diet and exercise in obese patients, aligning with findings by Farbod et al.^16^ and Jorge et al.^17^ who reported no significant resistin changes after similar interventions. However, Jung et al. observed decreases in BMI, cholesterol, TAG, and resistin after a 12-week diet/exercise program, supporting reports linking resistin reduction to weight loss. We found significant negative correlations between serum resistin and age, BMI, total cholesterol, and HbA1c, suggesting a relationship with obesity, hypercholesterolemia, and poor glucose control. Additionally, resistin positively correlated with weight loss, apelin, and SREBP-1c. The literature shows varying views on resistin's role in obesity; Savage et al. noted a higher resistin expression in obese adipose tissue, largely from inflammatory cells rather than adipocytes, suggesting mouse data on resistin may not directly apply to humans^18^.

Because obesity is considered a proinflammatory condition, serum levels of some cytokines in obese individuals may vary depending on the degree of weight loss. In our study, where BMI reductions ranged from 5 to 9%, no significant changes were observed in resistin levels after diet and exercise. In a study conducted in Korea by Jung et al., 78 obese patients experienced a 7% decrease in BMI and a significant decrease in resistin levels after a 3-month diet and exercise program^5^. In this study, which had a similar study protocol and sample size (serum) to ours, patients were additionally administered 10–15 mg of sibutramine. While resistin is also produced in adipose tissues in humans, the primary source of resistin is immune cells. Monocytes and macrophages are the primary sources of resistin in humans, and resistin is expressed heavily in PBMCs. Therefore, resistin acts more as an inflammatory adipokine in humans. Although the inclusion criteria in our study were similar to those of Jung et al., we lacked information on the patients’ immune status. Contrary to the literature, we did not observe the expected decrease in resistin levels after diet and exercise in our study. This may be due to biological variation caused by the population differences between the patients in the two studies, differences in their immune systems, the fact that patients in the literature study experienced easier weight loss with sibutramine, and the relatively small sample size in our study. Consequently, the role of resistin in obesity remains controversial, and we faced challenges interpreting our resistin findings within our study's limitations.

Unlike resistin, apelin supports insulin action. Its elevated levels in obesity may reflect a compensatory response, similar to insulin increases in insulin resistance. With an average weight loss of 8.4%, our findings align with previous studies: Sheibani et al. reported decreased apelin after 7.4% weight loss through exercise^19^, and Castan-Laurell et al. observed similar reductions in BMI (↓7.4%) following a low-calorie diet^20^. However, we have to point out that the lack of a nonintervention control group is a limitation of our study, as it raises the possibility of regression-to-the-mean or seasonal influences. Castan-Laurell et al. included 20 obese women (BMI: 32.2±6.4 kg/m^2^) and 12 healthy women (BMI: 20.7±0.6 kg/m^2^) as a control group in their study in which fasting plasma apelin levels were measured before and after a 3-month hypocaloric weight loss diet. It should be considered that, despite we determined reductions in apelin levels similar to the study conducted by Castan-Laurell et al., as a limitation, we could not blind or control the possible metabolic changes in the control group in the 3-month period.

It is still unclear whether the elevated levels of apelin seen in obesity are an attempt to overcome insulin resistance, obesity-related cardiovascular diseases, or another metabolic defect like apelin resistance, despite apelin being thought of as a beneficial adipokine that is upregulated in obesity. Thus, the reduction of apelin levels after weight loss should further be evaluated with insülin sensitivity indices, and it is crucial to comprehend how this adipokine contributes to problems linked to obesity.

Sterol regulatory element binding proteins-1c likely enter the bloodstream from cells involved in lipogenesis; when these cells lyse, the transcription factor is released. Although no human studies on serum SREBP-1c levels in obesity or postdiet/exercise changes were available, data from tissue studies provide insights. Ducluzeau et al. reported lower SREBP-1c mRNA in adipose tissues of diabetic and obese patients, with a negative correlation to BMI, supporting our serum findings^21,22^. Other studies showed increased SREBP-1c mRNA in insulin-resistant obese individuals and regional variations in adipose tissue expressions, which rose with weight loss^23^. Insulin is known to increase the SREBP1 gene expression. This is manifested by decreased transcript levels of SREBP1c, the most abundant isoform in adipose tissues, in obese normoglycemic and type 2 diabetic individuals compared to nonobese normoglycemic controls. Conversely, in skeletal muscles, the SREBP1 expression is significantly reduced in individuals with type 2 diabetes but not in obese individuals^24^. As is well known, the initial effects of obesity begin with hypertrophy and hyperplasia of fat cells. These hormones and cytokines secreted from adipose tissues play a role in the onset of insulin resistance, the chronicity of inflammation, and the basis of obesity and diabetes. The liver is the secondary organ in obesity due to the effects of free fatty acids and adipokines released from adipose tissues. Because the liver is the center of fatty acid and glucose metabolism, fat accumulation, deepening insulin resistance, and dyslipidemia become evident in the liver when obesity develops. Given that adipose tissue mass in the body is much greater than the liver, the inverse correlation of SREBP-1c levels with BMI observed in our study suggests that adipocytes are the primary source of obesity.

Considering that resistin is effective in increasing insulin resistance and SREBP-1c in increasing lipogenesis, it can be concluded that these correlations are important in the development of obesity. As explained in the introduction section, the transcription factor of the SREBP family that regulates cholesterologenesis is SREBP 2. Unfortunately, we could not measure serum SREBP-2 levels in our study. The change in serum total cholesterol levels in obesity could perhaps be explained by a congruent change in this transcription factor.

This study has the following limitations: A smaller number of cases than planned before the COVID-19 pandemic, unbalanced distribution of gender, failure to apply diet/exercise program for longer periods in obese patients, and lack of a control group. Initially, the study was planned for at least 1 year (four semesters); however, due to coinciding with the pandemic period, it could be carried out as a single 3-month period.

## CONCLUSION

A decrease in serum apelin levels was observed in the total obese group and in each of the three subgroups 3 months after the diet/exercise period, which can be interpreted as a kind of normalization from high preprogram levels. In addition, the significant positive correlation of apelin - BMI confirms the above-mentioned interpretation. A decrease in TAG and HbA1c was observed in the patients after the diet/ exercise program, which is natural in the case of weight loss and helpful for the patients. An increase in SREBP-1c levels was seen in obese subjects after the diet/exercise program. Based on the results of the correlation analysis of serum SREBP-1c levels, there are clues indicating that for obesity (based on BMI increase) and prediabetic/diabetic course (based on HbA1c), this transcription factor may be an important indicator.

## Data Availability

The datasets generated and/or analyzed during the current study are available from the corresponding author upon reasonable request.

## References

[1] Kirichenko TV, Markina YV, Bogatyreva AI, Tolstik TV, Varaeva YR, Starodubova AV (2022). The role of adipokines in inflammatory mechanisms of obesity. Int J Mol Sci.

[2] Zhou L, Li JY, He PP, Yu XH, Tang CK (2021). Resistin: potential biomarker and therapeutic target in atherosclerosis. Clin Chim Acta.

[3] Ghanbari M, Lamuki MS, Habibi E, Sadeghimahalli F, Artemisia annua L (2022). Extracts improved insulin resistance via changing adiponectin, leptin and resistin production in HFD/STZ diabetic mice. J Pharmacopuncture.

[4] Taouis M, Benomar Y (2021). Is resistin the master link between inflammation and inflammation-related chronic diseases?. Mol Cell Endocrinol.

[5] Jung SH, Park HS, Kim KS, Choi WH, Ahn CW, Kim BT (2008). Effect of weight loss on some serum cytokines in human obesity: increase in IL-10 after weight loss. J Nutr Biochem.

[6] Li C, Cheng H, Adhikari BK, Wang S, Yang N, Liu W (2022). The role of apelin-APJ system in diabetes and obesity. Front Endocrinol (Lausanne).

[7] Chapman FA, Maguire JJ, Newby DE, Davenport AP, Dhaun N (2023). Targeting the apelin system for the treatment of cardiovascular diseases. Cardiovasc Res.

[8] Boucher J, Masri B, Daviaud D, Gesta S, Guigné C, Mazzucotelli A (2005). Apelin, a newly identified adipokine up-regulated by insulin and obesity. Endocrinology.

[9] Kołodziejski PA, Pruszyńska-Oszmałek E, Wojciechowicz T, Sassek M, Leciejewska N, Jasaszwili M (2021). The role of peptide hormones discovered in the 21st century in the regulation of adipose tissue functions. Genes (Basel).

[10] Hu G, Wang Z, Zhang R, Sun W, Chen X (2021). The role of apelin/apelin receptor in energy metabolism and water homeostasis: a comprehensive narrative review. Front Physiol.

[11] Jiang SY, Yang X, Yang Z, Li JW, Xu MQ, Qu YX (2022). Discovery of an insulin-induced gene binding compound that ameliorates nonalcoholic steatohepatitis by inhibiting sterol regulatory element-binding protein-mediated lipogenesis. Hepatology.

[12] Elm E, Altman DG, Egger M, Pocock SJ, Gøtzsche PC, Vandenbroucke JP (2008). The strengthening the reporting of observational studies in epidemiology (STROBE) statement: guidelines for reporting observational studies. J Clin Epidemiol.

[13] Giordano MV, Alvarenga TF, Bastos CS, Giordano MG, Baracat EC, Soares JM (2021). Does obesity modify the expression of cyclin D1 and pten in endometrial polyps in postmenopausal women?. Gynecol Endocrinol.

[14] Cohen J (2013). Statistical power analysis for the behavioral sciences.

[15] Turri JAO, Anokye NK, Santos LL, Júnior JMS, Baracat EC, Santo MA (2022). Impacts of bariatric surgery in health outcomes and health care costs in Brazil: interrupted time series analysis of multi-panel data. BMC Health Serv Res.

[16] Farbod M, Eizadi M, Rashidi M, Mirakhori Z (2020). Effects of aerobic training with no caloric restriction on serum resistin and lipid profile in inactive overweight women. Int J Basic Sci Med.

[17] Jorge ML, Oliveira VN, Resende NM, Paraiso LF, Calixto A, Diniz AL (2011). The effects of aerobic, resistance, and combined exercise on metabolic control, inflammatory markers, adipocytokines, and muscle insulin signaling in patients with type 2 diabetes mellitus. Metabolism.

[18] Savage DB, Sewter CP, Klenk ES, Segal DG, Vidal-Puig A, Considine RV (2001). Resistin / Fizz3 expression in relation to obesity and peroxisome proliferator-activated receptor-gamma action in humans. Diabetes.

[19] Sheibani S, Hanachi P, Refahiat MA (2012). Effect of aerobic exercise on serum concentration of apelin, TNFα and insulin in obese women. Iran J Basic Med Sci.

[20] Castan-Laurell I, Vítkova M, Daviaud D, Dray C, Kováciková M, Kovacova Z (2008). Effect of hypocaloric diet-induced weight loss in obese women on plasma apelin and adipose tissue expression of apelin and APJ. Eur J Endocrinol.

[21] Ducluzeau PH, Perretti N, Laville M, Andreelli F, Vega N, Riou JP (2001). Regulation by insulin of gene expression in human skeletal muscle and adipose tissue. Evidence for specific defects in type 2 diabetes. Diabetes.

[22] Vidal H (2002). Regulation of gene expression in human skeletal muscle and adipose tissue. Ann Endocrinol (Paris).

[23] Kolehmainen M, Vidal H, Alhava E, Uusitupa MI (2001). Sterol regulatory element binding protein 1c (SREBP-1c) expression in human obesity. Obes Res.

[24] Sewter C, Berger D, Considine RV, Medina G, Rochford J, Ciaraldi T (2002). Human obesity and type 2 diabetes are associated with alterations in SREBP1 isoform expression that are reproduced ex vivo by tumor necrosis factor-alpha. Diabetes.

