# Supplementary material for: Relationship between obesity and serum resistin, apelin, and sterol regulatory element binding protein-1c levels: the changes in the analyte levels durin g weight loss in obese patients
Source: Rev Assoc Med Bras (1992). 2025 Dec 15;71(12):e20250751. doi: 10.1590/1806-9282.20250751 (PMC12711131; doi:10.1590/1806-9282.20250751)
Supplement: Supplementary Table 1 [file 1806-9282-ramb-71-12-e20250751-suppl1.docx]

**Supplementary Table 1**. Characteristics of the enzyme-linked immunosorbent assay kits.

|  | **Human resistin ELISA kit^1^** | **Human apelin ELISA kit^2^** | **Human SREBP-1c ELISA kit^3^** |
| --- | --- | --- | --- |
| Company | BT Lab, China | BT Lab, China | BT Lab, China |
| Cat. No | E0338Hu | E2014Hu | E6666Hu |
| Standard curve (detection) range | 20–6,000 ng/L | 7–1,500 ng/L | 0.2–60 ng/mL |
| Sensitivity | 10.2 ng/L | 3.47 ng/L | 0.15 ng/mL |
| Inter-assay precision (CV%) | <10% | <10% | <10% |
| Intra-assay precision (CV%) (based on three samples) | 3.7–4.9% | 2.9–6.6% | 3.4–5.6% |

Spike-and-recovery data and linearity-of-dilution assessments were not provided in the manufacturer’s datasheet. ELISA: enzyme-linked immunosorbent assay; SREBP-1c: sterol regulatory element binding protein-1c.

**REFERENCES**

1. Human Resistin ELISA kit (BT Laboratory) data sheet. Available from: https://www.bt-laboratory.com/index.php/Shop/Index/productList.html?kw=HUMAN+RESISTIN&_gl=1*1b8ktr1*_up*MQ..*_ga*MTcxMDU1NDEzNC4xNzU1NTAyNTQ1*_ga_N0R6VR6EMY*czE3NTU1MDI1NDIkbzEkZzEkdDE3NTU1MDI1NTkkajQzJGwwJGgw*_ga_LBGBHXB0YB*czE3NTU1MDI1NDQkbzEkZzEkdDE3NTU1MDI1NTkkajQ1JGwwJGgw*_ga_Q49XNJR62P*czE3NTU1MDI1NDIkbzEkZzEkdDE3NTU1MDI1NTgkajQ0JGwwJGgw*_ga_EWVMH18PXT*czE3NTU1MDI1NDEkbzEkZzEkdDE3NTU1MDI1NTkkajQyJGwwJGgxNzU3MjUzMjkz
2. Human Apelin ELISA kit (BT Laboratory) data sheet. Available from: <https://www.bt-laboratory.com/index.php/Shop/Index/productList.html?kw=HUMAN+APELIN&_gl=1*1n3yquv*_up*MQ..*_ga*MTcxMDU1NDEzNC4xNzU1NTAyNTQ1*_ga_N0R6VR6EMY*czE3NTU1MDI1NDIkbzEkZzAkdDE3NTU1MDI1NDIkajYwJGwwJGgw*_ga_LBGBHXB0YB*czE3NTU1MDI1NDQkbzEkZzAkdDE3NTU1MDI1NDQkajYwJGwwJGgw*_ga_EWVMH18PXT*czE3NTU1MDI1NDEkbzEkZzAkdDE3NTU1MDI1NDEkajYwJGwwJGgxNzU3MjUzMjkz*_ga_Q49XNJR62P*czE3NTU1MDI1NDIkbzEkZzAkdDE3NTU1MDI1NDIkajYwJGwwJGgw>
3. Human [Human Sterol regulatory element-binding protein 1C](https://www.bt-laboratory.com/index.php/Shop/Index/productShijiheDetail/p_id/26215.html?_gl=1*1jl0f13*_up*MQ..*_ga*MTcxMDU1NDEzNC4xNzU1NTAyNTQ1*_ga_LBGBHXB0YB*czE3NTU1MDI1NDQkbzEkZzEkdDE3NTU1MDI3NDQkajEzJGwwJGgw) (SREBP-1c) ELISA kit (BT Laboratory) data sheet. Available from: https://www.bt-laboratory.com/index.php/Shop/Index/productList.html?kw=HUMAN+SREBP-1c&_gl=1*1e9e13g*_up*MQ..*_ga*MTcxMDU1NDEzNC4xNzU1NTAyNTQ1*_ga_LBGBHXB0YB*czE3NTU1MDI1NDQkbzEkZzEkdDE3NTU1MDI2OTckajYwJGwwJGgw

**Supplementary Table 2.** The corelation analysis of all variables.

| **Correlations** | | | | | | | | | | | | | | |
| --- | --- | --- | --- | --- | --- | --- | --- | --- | --- | --- | --- | --- | --- | --- |
|  | | | **BMI (cm/kg)** | **% Weight loss** | **Cholesterol** | **HDL** | **LDL** | **Triglyceride** | **Glucose** | **Insulin** | **HbA1c** | **Resistin** | **Apelin** | **SREBP** |
| Spearman’s rho | BMI (cm/kg) | Correlation coefficient | 1.000 | 0.027 | 0.426** | -0.101 | 0.256** | 0.365** | 0.555** | 0.471** | 0.659** | -0.324** | 0.310** | -0.225* |
|  |  | Sig. (2-tailed) | . | 0.835 | 0.000 | 0.289 | 0.007 | 0.000 | 0.000 | 0.000 | 0.000 | 0.001 | 0.001 | 0.019 |
|  |  | n | 111 | 62 | 111 | 111 | 111 | 111 | 111 | 111 | 111 | 106 | 111 | 108 |
|  | % Weight loss | Correlation coefficient | 0.027 | 1.000 | -0.104 | -0.122 | -0.014 | -0.253* | -0.116 | -0.076 | -0.339** | 0.221 | -0.037 | 0.218 |
|  |  | Sig. (2-tailed) | 0.835 |  | 0.419 | 0.343 | 0.914 | 0.047 | 0.371 | 0.557 | 0.007 | 0.096 | 0.775 | 0.097 |
|  |  | n | 62 | 62 | 62 | 62 | 62 | 62 | 62 | 62 | 62 | 58 | 62 | 59 |
|  | Cholesterol | Correlation coefficient | 0.426** | -0.104 | 1.000 | 0.303** | 0.913** | 0.520** | 0.424** | 0.298** | 0.437** | -0.264** | 0.005 | -0.245* |
|  |  | Sig. (2-tailed) | 0.000 | 0.419 | . | 0.001 | 0.000 | 0.000 | 0.000 | 0.001 | 0.000 | 0.006 | 0.958 | 0.010 |
|  |  | n | 111 | 62 | 111 | 111 | 111 | 111 | 111 | 111 | 111 | 106 | 111 | 108 |
|  | HDL | Correlation coefficient | -0.101 | -0.122 | 0.303** | 1.000 | 0.222* | -0.319** | -0.016 | -0.052 | -0.066 | 0.017 | -0.003 | -0.011 |
|  |  | Sig. (2-tailed) | 0.289 | 0.343 | 0.001 |  | 0.019 | 0.001 | 0.868 | 0.585 | 0.493 | 0.866 | 0.975 | 0.911 |
|  |  | n | 111 | 62 | 111 | 111 | 111 | 111 | 111 | 111 | 111 | 106 | 111 | 108 |
|  | LDL | Correlation coefficient | 0.256** | -0.014 | 0.913** | 0.222* | 1.000 | 0.366** | 0.313** | 0.215* | 0.272** | -0.130 | -0.049 | -0.142 |
|  |  | Sig. (2-tailed) | 0.007 | 0.914 | 0.000 | 0.019 | . | 0.000 | 0.001 | 0.023 | 0.004 | 0.185 | 0.611 | 0.143 |
|  |  | n | 111 | 62 | 111 | 111 | 111 | 111 | 111 | 111 | 111 | 106 | 111 | 108 |
|  | Triglyceride | Correlation coefficient | 0.365** | -0.253* | 0.520** | -0.319** | 0.366** | 1.000 | 0.360** | 0.370** | 0.450** | -0.171 | 0.085 | -0.134 |
|  |  | Sig. (2-tailed) | 0.000 | 0.047 | 0.000 | 0.001 | 0.000 |  | 0.000 | 0.000 | 0.000 | 0.080 | 0.377 | 0.168 |
|  |  | n | 111 | 62 | 111 | 111 | 111 | 111 | 111 | 111 | 111 | 106 | 111 | 108 |
|  | Glucose | Correlation coefficient | 0.555** | -0.116 | 0.424** | -0.016 | 0.313** | 0.360** | 1.000 | 0.297** | 0.626** | -0.191 | 0.153 | -0.040 |
|  |  | Sig. (2-tailed) | 0.000 | 0.371 | 0.000 | 0.868 | 0.001 | 0.000 |  | 0.002 | 0.000 | 0.050 | 0.109 | 0.684 |
|  |  | n | 111 | 62 | 111 | 111 | 111 | 111 | 111 | 111 | 111 | 106 | 111 | 108 |
|  | Insulin | Correlation coefficient | 0.471** | -0.076 | 0.298** | -0.052 | 0.215* | 0.370** | 0.297** | 1.000 | 0.429** | -0.002 | 0.205* | -0.036 |
|  |  | Sig. (2-tailed) | 0.000 | 0.557 | 0.001 | 0.585 | 0.023 | 0.000 | 0.002 |  | 0.000 | 0.982 | 0.031 | 0.713 |
|  |  | n | 111 | 62 | 111 | 111 | 111 | 111 | 111 | 111 | 111 | 106 | 111 | 108 |
|  | HbA1c | Correlation coefficient | 0.659** | -0.339** | 0.437** | -0.066 | 0.272** | 0.450** | 0.626** | 0.429** | 1.000 | -0.378** | 0.085 | -0.362** |
|  |  | Sig. (2-tailed) | 0.000 | 0.007 | 0.000 | 0.493 | 0.004 | 0.000 | 0.000 | 0.000 |  | 0.000 | 0.374 | 0.000 |
|  |  | n | 111 | 62 | 111 | 111 | 111 | 111 | 111 | 111 | 111 | 106 | 111 | 108 |
|  | Resistin | Correlation coefficient | -0.324** | 0.221 | -0.264** | 0.017 | -0.130 | -0.171 | -0.191 | -0.002 | -0.378** | 1.000 | 0.181 | 0.723** |
|  |  | Sig. (2-tailed) | 0.001 | 0.096 | 0.006 | 0.866 | 0.185 | 0.080 | 0.050 | 0.982 | 0.000 |  | 0.063 | 0.000 |
|  |  | n | 106 | 58 | 106 | 106 | 106 | 106 | 106 | 106 | 106 | 106 | 106 | 105 |
|  | Apelin | Correlation coefficient | 0.310** | -0.037 | 0.005 | -0.003 | -0.049 | 0.085 | 0.153 | 0.205* | 0.085 | 0.181 | 1.000 | 0.205* |
|  |  | Sig. (2-tailed) | 0.001 | 0.775 | 0.958 | 0.975 | 0.611 | 0.377 | 0.109 | 0.031 | 0.374 | 0.063 | . | 0.034 |
|  |  | n | 111 | 62 | 111 | 111 | 111 | 111 | 111 | 111 | 111 | 106 | 111 | 108 |
|  | SREB | Correlation coefficient | -0.225* | 0.218 | -0.245* | -0.011 | -0.142 | -0.134 | -0.040 | -0.036 | -0.362** | **0.723**** | 0.205* | 1.000 |
|  |  | Sig. (2-tailed) | 0.019 | 0.097 | 0.010 | 0.911 | 0.143 | 0.168 | 0.684 | 0.713 | 0.000 | **0.000** | 0.034 | . |
|  |  | n | 108 | 59 | 108 | 108 | 108 | 108 | 108 | 108 | 108 | **105** | 108 | 108 |

**Correlation is significant at the 0.01 level (2-tailed). *Correlation is significant at the 0.05 level (2-tailed). BMI: body mass index; HDL: high-density lipoprotein; LDL: low-density lipoprotein; SREBP: sterol regulatory element binding protein.
